# Glycemic variability: prognostic impact on acute ischemic stroke and the impact of corrective treatment for hyperglycemia. The GLIAS-III translational study

**DOI:** 10.1186/s12967-020-02586-4

**Published:** 2020-11-04

**Authors:** Blanca Fuentes, Silvia Pastor-Yborra, Raquel Gutiérrez-Zúñiga, Noemí González-Pérez de Villar, Elena de Celis, Jorge Rodríguez-Pardo, Mari Carmen Gómez-de Frutos, Fernando Laso-García, María Gutiérrez-Fernández, MÁngeles Ortega-Casarrubios, Alfonso Soto, María López-Fernández, María Santamaría, Noemí Díez-González, Mar M. Freijo, Beatriz Zandio, Raquel Delgado-Mederos, Ana Calleja, Juan Carlos Portilla-Cuenca, Arturo Lisbona, Laura Otero-Ortega, Exuperio Díez-Tejedor

**Affiliations:** 1 Department of Neurology and Stroke Centre, Hospital La Paz Institute for Health Research-IdiPAZ (La Paz University Hospital, Universidad Autónoma de Madrid), Paseo de la Castellana 261, 28046 Madrid, Spain; 2 Department of Endocrinology, Hospital La Paz Institute for Health Research-IdiPAZ (La Paz University Hospital, Universidad Autónoma de Madrid), Paseo de la Castellana 261, 28046 Madrid, Spain; 3 Neurological Sciences and Cerebrovascular Research Laboratory, Department of Neurology and Stroke Centre, Neuroscience Area, Hospital La Paz Institute for Health Research-IdiPAZ (La Paz University Hospital, Universidad Autónoma de Madrid), Paseo de la Castellana 261, 28046 Madrid, Spain; 4 Department of Neurology, Hospital Universitario 12 de Octubre, Universidad Complutense de Madrid, Madrid, Spain; 5 Department of Endocrinology, Hospital Universitario A Coruña, A Coruña, Spain; 6 Department of Neurology, Hospital La Paz Institute for Health Research-IdiPAZ (La Paz University Hospital, Universidad Autónoma de Madrid), Santiago de Compostela, Spain; 7 Department of Neurology, Hospital de Donostia, San Sebastián, Spain; 8 Biocruces Bizkaia Health Research Institute, Department of Neurology, Hospital Universitario Cruces, Bizkaia, Spain; 9 Department of Neurology, Complejo Hospitalario de Navarra, Pamplona, Spain; 10 Department of Neurology, Hospital de la Santa Creu i San Pau, Barcelona, Spain; 11 Department of Neurology, Hospital Clínico Universitario de Valladolid, Valladolid, Spain; 12 Department of Neurology, Hospital San Pedro de Alcántara, Cáceres, Spain

**Keywords:** Ischemic stroke, Glycemic variability, Insulin, Outcomes, Translational research

## Abstract

**Introduction:**

Glycemic variability (GV) represents the amplitude of oscillations in glucose levels over time and is associated with higher mortality in critically ill patients. Our aim is to evaluate the impact of GV on acute ischemic stroke (IS) outcomes in humans and explore the impact of two different insulin administration routes on GV in an animal model.

**Methods:**

This translational study consists of two studies conducted in parallel: The first study is an observational, multicenter, prospective clinical study in which 340 patients with acute IS will be subcutaneously implanted a sensor to continuously monitor blood glucose levels for 96 h. The second study is a basic experimental study using an animal model (rats) with permanent occlusion of the middle cerebral artery and induced hyperglycemia (through an intraperitoneal injection of nicotinamide and streptozotocin). The animal study will include the following 6 groups (10 animals per group): sham; hyperglycemia without IS; IS without hyperglycemia; IS and hyperglycemia without treatment; IS and hyperglycemia and intravenous insulin; and IS and hyperglycemia and subcutaneous insulin. The endpoint for the first study is mortality at 3 months, while the endpoints for the animal model study are GV, functional recovery and biomarkers.

**Discussion:**

The GLIAS-III study will be the first translational approach analyzing the prognostic influence of GV, evaluated by the use of subcutaneous glucose monitors, in acute stroke.

*Trial registration* https://www.clinicaltrials.gov (NCT04001049)

## Introduction

Glycemic variability (GV) represents the amplitude of oscillations in blood glucose levels over time and has been associated with higher mortality and poor outcomes in critically ill patients [1, 2]. Few studies have evaluated the prognostic influence of GV in patients with acute ischemic stroke (IS) [3–8]; however, it has been suggested that GV is an even more powerful prognostic factor than poststroke hyperglycemia [9, 10]. Recently, the GLIAS-II study found higher GV in patients with acute IS who were considered nonresponders to conventional corrective treatment for hyperglycemia [11].

However, the factors influencing the development of wider GV in acute IS remain unknown. The use of sliding-scale subcutaneous insulin every 4–6 h has been suggested to be associated with large oscillations in blood glucose levels and, consequently, to higher GV [12]. Similarly, intensive intravenous insulin therapy has shown higher GV than standard treatment [6] and could explain the trend towards higher mortality in clinical trials aimed at a tight reduction in blood glucose levels in acute stroke [3].

One of the limitations of previous studies that evaluated GV is that it was measured with intermittent readings of blood glucose levels [5, 13], with inconsistent numbers of blood glucose measurements [14]. The development of continuous glucose monitors (CGM) [15, 16] has provided an opportunity for exploring the prognostic effect of GV in acute IS and those factors that affect the oscillations in blood glucose levels during the first days following a stroke.

We hypothesized that GV, assessable by means of continuous subcutaneous monitoring devices, could act as a powerful prognostic predictor of mortality, possibly higher than assessment of mean or maximum blood glucose levels and that the different treatment regimens used in routine clinical practice could modify glycemic variability. Our aim is to evaluate the impact of GV on IS outcomes and examine the impact of two different insulin administration routes on GV in an animal model of IS.

## Methods

The translational study consists of a multicenter observational study that will include patients with acute IS and a parallel basic experimental study using an animal model.

### Design

#### Clinical study

Observational, multicenter, prospective study.

##### Patient population

The inclusion and exclusion criteria are listed in Table 1.

**Table 1:** GLIAS-III main inclusion and exclusion criteria

Inclusion criteria	Exclusion criteria
Male and female patients older than 18 years, with acute IS Inclusion in the study within the first 24 h of stroke onset A prestroke score on the Modified Rankin Scale (mRS) ≤ 1 Signed informed consent	Transient ischemic attack Predicted need for an MRI within the first 96 h after inclusion A health status, clinical condition or other characteristic that precludes appropriate diagnosis, treatment, or follow-up in the trial Current drug or alcohol use dependence Participation in a therapeutic clinical trial

##### Randomization

None (observational study).

##### Intervention

After the participants sign the informed consent form, they will be implanted a CGM subcutaneously to monitor glucose levels for 96 h. These devices are of common use in the management of type 1 diabetes and they have been proven to be safe in stroke patients when used in research studies. Capillary finger prick glucose levels will be recorded every 4–6 h according to the standard protocols of each center, as well as the insulin administration characteristics (type, dose and route of administration) as per the treating physician’s criteria, following the local protocols for the in-hospital management of hyperglycemia. The treating physician and the nursing personnel will be blinded to the readings taken by the CMG device. All study centers will use the same brand of CGM (CE-marked). All patients will be managed according to current guidelines for acute stroke management and local protocols, including treatment with intravenous thrombolysis or mechanical thrombectomy if applicable. No laboratory data other than HbA1c will be collected for the study proposal. Any emergent laboratory abnormality during the study follow-up will be recorded as a complication.

##### Study schedule

The study will last 3 months per patient according to the schedule shown in Table 2.

**Table 2:** GLIAS-III flow diagram

	Baseline	96 h (± 6 h)	Day 7 or discharge (± 1 d)	Day 30 (± 2 d)		Day 90 (± 3 d)	
Signed informed consent	X						
Inclusion/exclusion criteria review	X						
Past medical/surgical history	x						
Pre-stroke treatments	x						
Modified rankin scale	(Pre-stroke)		X	X		X	
NIHSS	X	X	X	X		X	
Reperfusion treatment received	X						
HbA1c	X					X	
Continuous blood glucose monitoring	< –––––––––––––– >						
Capillary glucose levels (per local protocols)	< –––––––––––––– >						
Insulin (type, dosage and route) administered according to local protocols	< –––––––––––––– >						
Systemic and neurological complications	< ––––––––––––––––––––––––––––––––––––––––– >						
Stroke etiological subtype			X				
Secondary stroke prevention treatments			X		X		X

*HbA1c* glycated hemoglobin, *NIHSS*, National Institutes of Health Stroke Scale

##### Primary endpoints

- GV. Defined as the standard deviation of the mean blood glucose level for each patient [17]. For the main analysis, we will use the GV value recorded within the first 48 h after stroke onset. We will conduct an exploratory analysis to evaluate the GV between 48 and 96 h.
- Mortality: in-hospital and at 3 months.

##### Secondary endpoints

- Modified Rankin Scale (mRS) and National Institutes of Health Stroke Scale. (NIHSS) at 3 months. These scales will be evaluated by a neurologist blinded to the GV values.
- Other GV measurements: coefficient of variation (CV), mean amplitude of glycemic excursions (MAGE); mean absolute glucose (MAG) rate of change [2, 17, 18]. CV is less influenced by mean glucose and is a good marker of hypoglycemia. A threshold value to define low and high GV using CV has recently been proposed [19]. MAGE and MAG are indexes used for studying glycemic variability in clinical trials, and normal references values for people without diabetes using CGM are available [18]. On the other hand, MAGE can provide information about the influence of the largest glycemic excursions.
- Neurological or systemic complications during follow-up. The following complications are pre-specified by protocol and will be checked at each visit: coma, seizures, early neurological impairment, brain edema, hemorrhagic transformation, recurrent strokes, acute coronary syndrome, pulmonary thromboembolism, respiratory infection, urinary infection, sepsis. Any other non-pre-specified complication reported during follow-up will be also recorded.

##### Data collection and management

In addition to the primary and secondary endpoints, the following data will be collected:

- Demographic data and risk factors: age, sex, race, weight, height, diabetes, hypertension, dyslipidemia, coronary arterial disease, atrial fibrillation, metabolic syndrome, chronic renal disease (eGFR of less than 60 ml/min/1.73m2), tobacco use and alcohol abuse.
- Pre-stroke pharmacological treatments: antiplatelets, anticoagulants, blood-pressure lowering drugs, antidiabetics and hypolipemiants.
- Stroke data: stroke severity on admission (NIHSS), date of stroke onset (or last time known asymptomatic in those patients with unknown stroke onset), stroke etiological subtype, treatment with intravenous thrombolysis or mechanical thrombectomy.
- Vital signs at baseline visit: body temperature, blood pressure, heart rate, respiratory rate.

Data will be prospectively included in a study-specific web-based database developed and managed by the Clinical Trials Unit at Hospital 12 de Octubre which belongs to the Spanish Clinical Research Network (SCReN), independent form the study sponsor. All data management will follow the principles of the European regulations for biomedical research ensuring confidentiality. In compliance with European regulations/International Conference of Harmonization Good Clinical Practice Guidelines, the investigator and the institution are required to permit direct access to authorized representatives of the Ethics Committee to review the subject’s original medical records for verification of study-related procedures and data.

##### Data monitoring body

Monitoring will be conducted by dedicated personnel at La Paz University Hospital. All data management will follow the principles of the European regulations for biomedical research, ensuring the confidentiality of all personal data. In compliance with European regulations/International Conference of Harmonization Good Clinical Practice (ICHGCP) Guidelines, the investigator and institution are required to permit authorized representatives of the regulatory agency(s), and the IEC/IRB direct access to review the subject’s original medical records for verification of study-related procedures and data.

##### Sample size estimates

Based on the data from the GLIAS-II study, [11] conducted in a similar setting and using nQuery Advisor software (Statistical Solutions Ltd., Cork, Ireland), we calculated that a sample of 340 patients will be required to achieve 80% statistical power and a confidence level alpha of 0.05 to detect a significant effect of GV on mortality at 3 months.

##### Recruiting hospitals

Hospital Universitario La Paz (Madrid), Hospital Universitario 12 de Octubre (Madrid), Hospital Universitario A Coruña (A Coruña), Hospital Universitario de Santiago de Compostela (Santiago de Compostela), Hospital de Donostia (San Sebastián), Hospital Universitario Cruces (Bizkaia), Complejo Hospitalario de Navarra (Pamplona), Hospital de la Santa Creu i San Pau (Barcelona), Hospital Clínico Universitario de Valladolid (Valladolid) and Hospital San Pedro de Alcántara (Cáceres).

#### Experimental animal model of stroke and hyperglycemia in rats

The basic experimental study will use an animal model of permanent middle cerebral artery occlusion (MCAO) and hyperglycemia in rats. The experiments are designed to minimize animal suffering in compliance with our Ethical Committee for the Care and Use of Animals in Research (EU directives 86/609/CEE and 2003/65/CE).

##### Study groups

A total of 60 Sprague–Dawley rats (8–9 weeks old, weighing 200–250 g) will be randomly assigned to the following groups:

1. Sham: hyperglycemia and surgery without MCAO.
2. Non-stroke group: hyperglycemia without MCAO.
3. Normoglycemic group: normoglycemia with MCAO.
4. Non-treated hyperglycemic group: hyperglycemia with MCAO.
5. Intravenously treated hyperglycemic group: Hyperglycemia with MCAO and intravenous insulin administration (dosage, 3 U/day).
6. Subcutaneously treated hyperglycemic group: Hyperglycemia with MCAO and subcutaneous insulin administration (dosage, 3 U/day).

##### Randomization

We will follow RIGOR recommendations for animal research in terms of randomization and blinded study [20]. Each rat will be sequentially assigned to a study group based on the randomization plan. To maintain the blinding during the course of the study, we will ensure that the researcher who evaluates the endpoints and performs the laboratory analyses will not have access to the randomization codes, and a different researcher will perform the surgery, hyperglycemia induction and insulin administration. Following the STAIR recommendations, both female and male rats will be included (1:1 ratio) [21]. The stage of estrous cycle will be recorded. If variability is observed in the final results, a specific post-hoc analysis of the estrous cycle stage will be performed.

##### Intervention

Hyperglycemia will be induced by an intraperitoneal injection of nicotinamide (210 mg/kg) (EMD Millipore, Germany). Fifteen minutes later, an intraperitoneal injection of streptozotocin (60 mg/kg) (EMD Millipore, Germany) will be administered [22, 23]. Blood glucose levels will then be measured using a glucometer (ACCU-CHEK, Performa, Germany) for 72 h. The animals with a blood glucose level > 250 mg/dL will be considered hyperglycemic [23].

To induce a stroke, the rats will be anesthetized via an intraperitoneal injection of a solution of ketamine (25 mg/kg) and diazepam (2 mg/kg). Analgesia will be induced with a subcutaneous injection of meloxicam (2 mg/kg). To perform the MCAO, a small craniotomy will be performed, and the right middle cerebral artery will be permanently ligated just before its bifurcation. Both common carotid arteries will then be occluded for 60 min, as previously described [24]. A device for CGM will then be implanted subcutaneously for at least 96 h.

Insulin treatment will be administered 4 h after MCAO and until 96 h later. The administration will be intravenous or subcutaneous, depending on the experimental group. The dosage per animal (3 U/day) has been determined based on the results of a previous dose–response study [23].

##### Study schedule

**Fig. 1:**
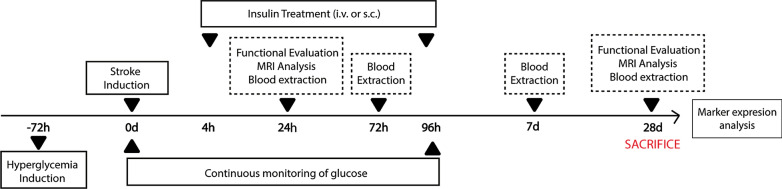
Experimental animal protocol. Hyperglycemia will be induced 72 h before surgery. The rats will then undergo a IS through permanent middle cerebral artery occlusion or sham surgery. Insulin treatment will be intravenously or subcutaneously administered 4 h after IS induction and up to 96 h later, after which the blood glucose levels will be continuously monitored. A functional evaluation will be performed at 24 h, 1 week and 4 weeks post-stroke. Peripheral blood will be extracted 24 h, 72 h and 4 weeks after the stroke. The MRI will be analyzed at 24 h and 4 weeks post-stroke. Four weeks after the stroke, the animals will be euthanized, and histological analyses will be performed

The study will last a total of 31 days for each rat, according to the schedule illustrated in Fig. 1.

##### Primary endpoint

- GV, defined as the standard deviation of the mean glucose level [17]. We will analyze whether the insulin administration route (intravenous and subcutaneous) affects the GV.

##### Secondary endpoints

- Functional evaluation, analyzed by a researcher blinded to the experimental groups, at 24 h, 1 week and 4 weeks post-stroke. Motor and sensory performance will be evaluated using a variant of Rogers’ functional scale [23]. The beam walking test will evaluate hindlimb functions by testing the rats’ ability to traverse a wooden beam [23].
- Lesion size will be analyzed at 24 h and 4 weeks post-stroke by 7-T horizontal bore magnetic resonance imaging (MRI) (Bruker PharmaScan, Ettlingen, Germany), using T2-weighted (T2-W) spin-echo anatomical images, by a researcher who will be blinded to the experimental groups.
- Brain damage and repair-related markers will be analyzed at 4 weeks. The perilesional area of the brain will be studied in detail using immunofluorescence for superoxide dismutase 2, catalase, vascular endothelial growth factor (VEGF), brain-derived neurotrophic factor (BDNF), synaptophysin, glial fibrillary acidic protein, myelin basic protein and oligodendrocyte. Immunofluorescence images will be acquired by a researcher (who will be blinded to the experimental groups) using a Leica TCS-SPE confocal microscope (Leica Microsystems, Heidelberg, Germany). Plasma levels of damage and repair markers will be studied using Multiplex (eBioscience) and by ELISA (System Biosciences, Mountain View, CA, USA). The following markers will be analyzed: interleukin-1, interleukin-6, interleukin-10, vascular cell adhesion molecule, tumor necrosis factor alpha, matrix metalloproteinase 9, VEGF, BDNF, anti-Nogo A.

##### Sample size estimates

Based on data from previous studies [11, 25] and using the statistical software NCSS PASS 11 (NCSS, Utah, USA), we calculated that a sample of 10 rats per group will be required to achieve a power of 80% (1-beta) and a level of significance of 5% (alpha) to detect a significant effect of GV on the study endpoints.

### Statistical analysis

The statistical analysis will be performed with advice from the Department of Biostatistics of La Paz University Hospital. ﻿For the continuous variables, the following information will be provided: number of participants, mean, standard deviation, median, minimums, maximums and 25% and 75% quartiles. For the categorical variables, the frequency distribution and 95% confidence interval (CI), if applicable, will be provided. The analysis of the prognostic influence of GV on stroke outcomes will be performed in an exploratory manner without assumptions. We will first analyze GV as a continuous variable, comparing it with primary and secondary endpoints using Student’s t-test or the Mann–Whitney U test, as appropriate. We will also compare GV according to the various insulin types and administration routes applied to the patients according to the physician’s discretion. The comparisons will be performed with the one-way analysis of variance test. Secondly, we will perform a step forward logistic regression analysis for the endpoints that achieved a difference with a *P* value < 0.1 in the mean comparison tests, adjusting for age and baseline National Institutes of Health Stroke Scale score. The results will be expressed as odds ratios (OR) with 95% CI. In case of lack of homogeneity in the variances, we will use non-parametric tests (Kruskal–Wallis and Mann–Whitney test).

## DIscussion

To our knowledge, few studies have used continuous glucose monitors in acute IS [7, 8, 26–28]. A relationship has been found between GV (by means of the standard deviation of mean glucose levels) and infarct growth [8], as well as between GV and early neurological deterioration [29]. The main limitations of the prior studies using CGM in acute stroke are their monocentric settings, the lack of analysis of the effect of insulin administration on GV and the heterogeneity in the index used to express GV, given there is no widely accepted optimal index [30]. The GLIAS-III study is designed to overcome those limitations, with a prospective and multicenter approach, and is aimed at recruiting more than 300 patients. Moreover, the development of a parallel preclinical study using an animal model of permanent MCAO will increase our knowledge of the effect of insulin administration on GV, functional outcomes, lesion size, brain damage and repair-related markers.

## Data Availability

After the completion of the trial, raw data will be deposited in an institutional repository and final results will be published in Open Access journals.
